# Factors associated with interruption in treatment among pregnant and breastfeeding women living with HIV on ART in South Sudan

**DOI:** 10.1186/s12889-026-27413-1

**Published:** 2026-04-15

**Authors:** Silvestro Ojja, Amy Benson, Megan Swanson, Elizabeth Carter, Katherine A Battey, Sarah Auma, Akech Awan, Alex Bolo, Michele Montandon, Sudhir Bunga

**Affiliations:** 1 Division of Global HIV & Tuberculosis, Global Health Center, Centers for Disease Control and Prevention Juba, Juba, South Sudan; 2 National Ministry of Health, Juba, South Sudan; 3 Division of Global HIV & Tuberculosis, Center for Global Health, Centers for Disease Control and Prevention Atlanta, Atlanta, Georgia

**Keywords:** Interruption in treatment, continuity in treatment, retention in HIV care, MMD among PBFW

## Abstract

**Background:**

Continuity of HIV treatment among pregnant and breastfeeding women (PBFW) living with HIV is challenging in South Sudan. Understanding factors associated with interruption in treatment (IIT) among PBFW on antiretroviral therapy (ART) may inform programmatic interventions to improve care and continuity of treatment.

**Methods:**

A retrospective cohort analysis of records of PBFW on ART in twenty health facilities was conducted. A probability-proportionate-to-size sampling (PPS) method was used to select facilities with PBFW on ART for at least six months from 01/10/2019 to 01/02/2022 prior to date of data abstraction. Demographic and clinical information were abstracted from facility-based registers and client files. Abstracted ART visit data were used to calculate IIT, and multi-month dispensing (MMD) of ART. Prevalence ratios (PRs) were used to calculate measures of association for IIT.

**Results:**

A total of 1,478 PBFW on ART were included; 37% had IIT. Home delivery compared to facility delivery (Adj.PR = 1.3, 95% C.I: 1.04–1.49) and receiving MMD for < 3 months compared to ≥ 6 months (Adj.PR = 1.6, 95% C.I: 1.30–2.00) were significantly associated with IIT among PBFW. Those attending one Antenatal Care (ANC) visit compared to ≥ 3 (Adj.PR = 0.8, 95% CI: 0.66–0.96) were less likely to have IIT.

**Conclusions:**

Over one-third of PBFW on ART had IIT, which was associated with three factors: number of ANC visits, place of delivery, and frequency of MMD. MMD for PBFW was not associated with IIT, supporting its use in areas with similar challenges to South Sudan.

**Supplementary Information:**

The online version contains supplementary material available at 10.1186/s12889-026-27413-1.

## Background

Ensuring continuity in HIV treatment and adherence to antiretroviral therapy (ART) throughout the pregnancy and postpartum periods is essential both for maternal health and to decrease the risk of vertical transmission. While prevention of vertical transmission (PVT) programs have greatly reduced new child infections over the past twenty years, *g*aps exist within the PVT cascade of services in South Sudan [1].

Interruptions in HIV treatment (IIT) during pregnancy and breastfeeding lead to increased maternal viral load, increased risk of mortality, HIV disease progression, and increased risk of vertical transmission, and potentially undiagnosed and untreated children living with HIV [2–4]. Estimates of continuity in HIV treatment among PBFW in sub-Saharan Africa range widely from 56% to 97% at the first PVT follow-up visit and from 47% to 88% 6 months after ART initiation [5]. High IIT rates have been described across all timepoints in the pregnant and postpartum periods [6].

In studies in Eastern and Southern Africa, low retention in HIV care and treatment during pregnancy and postpartum has been associated with rural residency, lower educational level, unemployment, non-disclosure of HIV status to a partner, age, newly initiating treatment, and late presentation to antenatal care [7–9].

Given the unique geo-political and health system context in South Sudan, it is important to understand if the same factors also contribute to IIT. South Sudan is the world’s youngest nation, and since gaining independence in 2011, remains impacted by political instability, conflict, and severe droughts and floods [10]. Many residents of South Sudan have poor access to health services and the maternal and infant mortality rates are among the highest in the world [11]. About 31% of women in South Sudan attended 4 + antenatal care (ANC) visits [12] compared to 58% of women in Sub-Saharan Africa [13], and an estimated 42% of women delivered in a health facility during their last pregnancy [12].

South Sudan is one of the few countries in the Eastern and Southern Africa region where the number of new child HIV infections is greater than the estimated new infections averted due to PVT programming [1]. In 2023, an estimated 6,200 pregnant women in South Sudan needed ART, with about 76% of those women receiving ART during pregnancy, and the vertical transmission rate of HIV was about 20% [1]. South Sudan prescribes 3- to 6-month refills of ART to PBFW as a standard of care. Multi-month dispensing (MMD) is a model that provides stable clients on ART with drug refills every 3 or 6 months instead of monthly to reduce ART clinic visits and support client-centered care. The model is supported by the World health Organization (WHO) for the general population [14]. Recognizing that conflict and frequent environmental disasters create potential difficulties for clients to access ART clinics, South Sudan’s national guidelines endorse dispensing ART for up to a 6- month supply, including to pregnant and breastfeeding women (PBFW) [15]. Given the unique context of South Sudan and the importance of treatment continuity in the PVT cascade, we aim to describe the factors associated with IIT among PBFW across the U.S. President’s Emergency Plan for AIDS Relief (PEPFAR)-supported sites in South Sudan.

## Methods

### Study setting and design

A retrospective cohort study was conducted from 01/08/2022 to 30/09/2022 using health facility-based records from 20 PEPFAR-supported health facilities in three urban (Juba, Yambio, and Torit), three peri-urban (Yei, Wau, and Rumbek), and four rural (Yirol West, Kapoeta South, Magwi, and Maridi) counties in South Sudan. The 10 counties were selected based on a high volume of PBFW on ART. Together, the 10 counties cover 80% of PBFW on ART among PEPFAR-supported facilities. PBFW in most of the PEPFAR-supported health facilities in South Sudan receive antenatal care (ANC) in a separate clinic from where they receive HIV care and ART; PBFW are tested for HIV at ANC clinics and if found to be positive, enrolled in an ART clinic and referred to PVT services. In South Sudan, PVT services include pre-and post-test counseling at ANC clinics, client treatment, viral load testing and treatment literacy during ART initiation and follow up at the ART and PVT clinics. While the clinics are usually in proximity, pregnant women living with HIV (PWLHIV) are required to see two separate providers, one to provide ANC and another to provide ART.

This was a secondary data analysis reviewed by CDC and was conducted consistently with applicable federal law and CDC policy. † Consent to participate was waived by the South Sudan National Ministry of Health research ethics review board (MOH-RERB).

### Study population

For this study, inclusion criteria include PBFW living with HIV, ages 14–45 years who were enrolled at selected health facilities providing PVT care, and on ART at the beginning of the study period. Eligible clients were pregnant and breastfeeding women living with HIV (PBFWLHIV) enrolled in ART clinics for at least six months from 01/10/2019 to 01/02/2022 prior to date of data abstraction. We excluded those who were missing data on date of enrollment.

### Sample size determination and data collection procedure

The following data sources were used to collect client-level information from facility records: (1) ANC PVT patient register to collect clients’ socio-demographic, gravidity, and ANC attendance data (2) pharmacy dispensing logs/ART register/missed appointment and IIT tracking registers to collect ART initiation, (3) Maternity register to collect data on place of delivery (4) HIV care and ART card to collect ART attendance data. Records for each patient were matched using a unique identifier.

Health facilities with PVT services were selected using probability-proportionate-to-size sampling (PPS). The sample size for the current study was informed by the duration of data abstraction. Records were utilized to assess IIT among PBFW living with HIV enrolled in the PVT program for at least six months from 01/10/2019 to 01/02/2022 prior to date of data abstraction.

which varied by facility and ranged from 01/08/2022 to 30/09/2022. Data were abstracted from hard copy documents/registers and to hard copy standardized study data collection forms that were stored in a secured location. Data from the study forms were entered into a secure electronic database designed in EPI-INFO (version 7.2.3.1, Centers for Disease Control and Prevention, Atlanta GA, USA).

### Study variables and measurement

The outcome variable was “interruption in treatment”, defined as missing the first ART follow-up visit after enrollment in PVT care by greater than 28 days from the scheduled date. IIT was measured as either ‘yes’ (missing first ART follow-up visit by greater than 28 days) or ‘no’ (documented first visit ART follow-up visit date is within 28 days of scheduled appointment date). This includes already on ART and new on ART clients. Data on sociodemographic, obstetric, and HIV-related variables was collected: maternal age at ANC registration, client location/residence, marital status, main occupation, parity, gravida, number of ANC visits, gestational age at ANC registration, HIV status at entry to ANC1 (known or new), HIV treatment status at entry to PVT (already on ART or newly initiating), and place of delivery. Multi-month drug dispensing (MMD) was calculated using the number of days between the initial visit date and the first scheduled follow-up visit date as a proxy to determine MMD. Those with a scheduled follow-up that was before the initial visit date were set as missing for the MMD variable. A month was defined as 30 days and categories were created for < 3 months (no MMD), 3–5 months, and *≥* 6 months.

### Statistical analysis

The data were entered into an automated database designed using EPI-INFO. Data was analyzed using STATA^®^ standard edition version 17 (Stata Corp LP, College Station, Texas, USA). Descriptive statistics were conducted to describe the demographic, obstetric, and HIV treatment characteristics of PBFWLHIV. The Pearson chi-square test was used to test for differences between groups. The differences between IIT and age, place of delivery, client residential area, and MMD were measured using Bartlett’s test for equal variances.

Prevalence ratios (PRs) were used as a measure of association between IIT and socio-demographic and other variables. A generalized linear model (GLM) analysis was conducted using a robust standard error Poisson regression, taking into consideration the high prevalence outcome. All independent factors with a p-value < 0.15 and biologically plausible variables were included in the multivariable model. A stepwise model-building technique was used to ascertain the best-fitting model with a log likelihood tending towards zero. For all tests, a p-value ≤ 0.05 at 95% confidence intervals (95% CI) was considered statistically significant. Unadjusted PRs (PRs), adjusted prevalence ratios (Adj.PR), and their respective 95% CI were reported.

## Results

### Socio-demographic characteristics

A total of 1,478 PBFW on ART were included in the study. Approximately one third resided in each of the urban, peri-urban, and rural areas settings (33.8%, 33.6%, and 32.6%, respectively). More than half of the participants (58.9%) were aged 20–29 years, with a median age of 27 years withan age range of 14–45. Majority (84.3%) of the participants were married and unemployed (80.1%). Most women (90.7%) were multigravida, 23.6% delivered at home, 31.8% enrolled in ANC before 16 weeks of gestational age, 53.2% had known HIV positive status before ANC enrollment, and 59.8% of all clients received ≥ 6 months of MMD (Table 1).

**Table 1:** Characteristics of PBFW on ART in 20 PEPFAR-supported Health Facilities, South Sudan, 2022

Variable	Frequency			Percentage (%)
Age at ANC Registration (*n* = 1474)				
14–19	113			7.7
20–29	868			58.9
30–39	475			32.2
40–45	18			1.2
Client’s Location/Residence (*n* = 1478)				
Urban area	500			33.8
Peri-Urban area	496			33.6
Rural area	482			32.6
Marital Status (*n* = 1410)				
Never Married	180			12.8
Married	1,188			84.3
Divorced/Widowed	42			2.9
Client’s Main Occupation (*n* = 1434)				
Unemployed	1,149			80.1
Employed	285			19.9
Client’s Gravida (Number of Pregnancies) (*n* = 1238)				
1	115			9.3
2	235			19.0
≥3	888			71.7
Number of ANC Visits (*n* = 1303)				
1 visit	639			49.1
2 visits	373			28.6
≥3 visits	291			22.3
Place of Delivery (*n* = 1397)				
At Health Facility	1,067			76.4
At home	330			23.6
Gestational age at ANC registration (*n* = 1170)				
<16 weeks	372			31.8
≥16 weeks	798			68.2
HIV status at entry to ANC 1 (*n* = 1430)				
Newly tested Positive	675			46.1
Known Positive	790			53.9
Birth Outcomes (*n* = 1382)				
Live Birth-LB	1,356			98.1
Still Birth-MSB	16			1.2
Neonatal Death-ND	10			0.7
Treatment status at entry to PVT (*n* = 1431)				
Already on ART	781			54.6
New on ART	650			45.4
MMD (*n* = 1,434)				
<3 Months	156			10.9
3–5 Months MMD	421			29.3
≥ 6 Months MMD	857			59.8

### Interruption in Treatment (IIT)

The overall proportion of clients with IIT was 37.2% (*n* = 550). Statistically significant differences were observed in treatment interruption among PBFW across marital status (*p* < 0.001), place of delivery (*p* = 0.012), number of ANC visits (*p* = 0.043), and MMD (*p* < 0.001). There were no differences in IIT by age, client’s location, or occupation (Table 2).

**Table 2:** The distribution of Interruption in Treatment Among Pregnant and Breastfeeding Women (PBFW) on ART in 20 PEPFAR-supported Health Facilities -South Sudan, 2022

					Treatment Interruption			
Maternal Factors	Frequency (*N*)		Yes (%)				No (%)	*p* (< 0.05) *
**Total**	1478		550 (37.2)				928 (62.8)	*N/A*
Age at ANC Registration								
14–19	113		38 (33.6)				75 (66.4)	***0.409***
20–29	868		323 (37.2)				545 (62.8)	
30–39	475		182 (38.3)				293 (61.7)	
40–45	18		6 (33.3)				12 (66.7)	
Total	1,474		549 (37.2)				925 (62.8)	
Client’s Location/Residence								
Urban area	500		204 (40.8)				296 (59.2)	***0.096***
Peri-Urban area	496		170 (34.3)				326 (65.7)	
Rural area	482		176 (36.5)				306 (63.5)	
Total	1,478		550 (37.2)				928 (62.8)	
Marital Status								
Never Married	180		48 (26.7)				132 (73.3)	***< 0.001***
Married	1,188		471 (39.7)				717 (60.3)	
Divorced/Widowed	42		10 (23.8)				32 (76.2)	
Total	1,410		529 (37.5)				881 (62.5)	
Client’s Main Occupation								
Unemployed	1,149		438 (38.1)				711 (61.9)	***0.166***
Employed	285		96 (33.7)				189 (66.3)	
Total	1,434		534 (37.2)				900 (62.8)	
Number of ANC Visits								
1 visit	639		224 (35.1)				415 (64.9)	***0.043***
2 visits	373		143 (38.3)				230 (61.7)	
3 + visits	291		127 (43.6)				164 (56.4)	
Total	1,303		494 (37.9)				809 (62.1)	
Place of Delivery								
At Health Facility	1,067		390 (36.6)				677 (63.4)	***0.012***
At home	330		128 (38.8)				202 (61.2)	
Total	1,397		518 (37.1)				879 (62.9)	
MMD								
< 3 months	156		75 (48.1)				81 (51.9)	***< 0.001***
3–5 Months MMD	421		177 (42.0)				244 (58.0)	
≥ 6 Months MMD	857		283 (33.0)				574 (67.0)	
Total	1,434		535 (37.3)				899 (62.7)	

*chi square test was conducted for p values

### Multivariable analysis of socio-demographic factors associated with interruption in treatment among PBFW on ART

After adjusting for potential confounders, the study found significant associations between ANC attendance, place of delivery, and MMD (Table 3). Pregnant women attending only one ANC visit were less likely to have ITT compared to those attending three or more ANC visits (Adj.PR = 0.8, 95% CI: 0.66–0.96). Women who delivered at home were statistically significantly more likely to have IIT compared to those who delivered at a health facility (Adj.PR = 1.3, 95% CI: 1.04–1.49). PBFW who received less than three months of medication were more likely to have IIT compared to those who received ≥ 6 months of medication (Adj.PR = 1.6, 95% CI: 1.30–2.00).

Client age at ANC registration, residence, HIV status at entry to ANC, main occupation, marital status, and treatment status at PVT enrollment were not significantly associated with treatment interruption among PBFW.

**Table 3:** Multivariate analysis of Potential Sociodemographic Factors Associated with Treatment Interruption among PBFW ART in 20 PEPFAR-Supported Health Facilities, South Sudan, 2022

		Interruption in Treatment				
**Variable**	**Total N**	**Yes [n (%)]**	**No [n (%)]**	**Unadjusted PR**,** 95% CI**	Adjusted PR^*|^, **95% CI**	P value
Age at ANC Registration (*N* = 1,474)						
14–19	113	38 (33.6)	75 (66.4)	0.9 [0.69–1.19]	1.0 [0.74–1.34]	0.972
20–29	868	323 (37.2)	545 (62.8)	**1**	**1**	
30–39	475	182 (38.3)	293 (61.7)	1.0 [0.89–1.19]	1.1 [0.95–1.32]	0.161
40–45	18	6 (33.3)	12 (66.7)	0.9 [0.46–1.73]	0.8 [0.34–1.69]	0.503
Client’s Location/Residence ,(*N* = 1478)						
Urban Area	500	204 (40.8)	296 (59.2)	**1**	**1**	
Peri-Urban Area	496	170 (34.3)	326 (65.7)	0.8 [0.72–0.99]	1.0 [0.79–1.19]	0.761
Rural Area	482	176 (36.5)	306 (63.5)	0.9 [0.76–1.05]	0.9 [0.73–1.08]	0.227
Marital Status (*N* = 1,410)						
Married	1,188	471 (39.7)	717 (60.3)	**1**	**1**	
Never Married	180	48 (26.7)	132 (73.3)	0.7 [0.52–0.87]	0.8 [0.59–1.04]	0.095
Divorced/Widowed	42	10 (23.8)	32 (76.2)	0.6 [0.35–1.04]	0.7 [0.39–1.21]	0.195
Number of ANC Visits (*N* = 1,303)						
3 + Visits	291	127 (43.6)	164 (56.4)	**1**	**1**	
2 Visits	373	143 (38.3)	230 (61.7)	0.9 [0.73–1.06]	0.9 [0.72–1.08]	0.217
1 visit	639	224 (35.1)	415 (64.9)	0.8 [0.68–0.95]	0.8 [0.66–0.96]	**0.019**
Place of Birth Delivery (*N* = 1,397)						
At Health Facility	1,067	390 (36.6)	677 (63.4)	1	1	
At home	330	128 (38.8)	202 (61.2)	1.1 [0.91–1.24]	1.3 [1.04–1.49]	**0.018**
Types of entry to PVT (*N* = 1,465)						
Known Positive †	790	310 (39.2)	480 (60.8)	0.9 [0.78–1.02]	1.0 [0.76–1.40]	0.850
Newly tested Positive ‡	675	235 (34.8)	440 (65.8)	1	**1**	
Treatment Status at Entry to PVT (*N* = 1,431)						
Already on ART ^§^	781	302 (38.7)	479 (61.3)	1	**1**	
New on ART ^||^	650	226 (34.8)	424 (65.2)	0.9 [0.78–1.03]	0.9 [0.68–1.26]	0.637
Clients’ Occupation (*N* = 1,434)						
Unemployed	1,149	438 (38.1)	711 (61.9)	1	1	
Employed	285	96 (33.7)	189 (66.3)	0.9 [0.74–1.06]	1.0 [0.82–1.22]	0.992
MMD (*N* = 1,434)						
<3 Months (No MMD)	156	75 (48.1)	81 (51.9)	1.5 [1.21–1.76]	1.6 [1.30–2.00]	**< 0.001**
3–5 Months	421	177 (42.0)	244 (58.0)	1.3 [1.10–1.48]	1.3 [1.12–1.58]	0.001
≥6 Months	857	283 (33.0)	574 (67.0)	1	1	

*PR *Prevalence Ratio,* CI C*onfidence interval

*p < 0.05;

† *(Newly tested positive at ANC 1 and Positive at Post ANC1)*

‡ *(Known positive on ART+ Known Positive not on ART)*

^§^ *(Already on ART prior to current ART enrolment*

^||^ *(New on ART in pregnancy and new on ART-postpartum)*

## Discussion

This study highlights the factors associated with interruption in treatment among PBFW enrolled in PVT in South Sudan. It demonstrates potential areas for improving the continuity of treatment for PBFW on ART. South Sudan is the youngest nation in the world, with multiple obstacles to the successful HIV treatment of PBFW. Implementing a PVT program in South Sudan is particularly challenging due to a combination of weak health infrastructure, and ongoing conflicts. Conflict and displacement have disrupted health systems and made large portions of the country inaccessible, especially during the rainy season [10]. Low ANC attendance and high rates of home births limit opportunities for HIV testing and maternal ART initiation [12].

Our analysis examined the following factors among PBFW: rural residency, education levels, employment, age, newly initiating HIV treatment, timing of presentation to antenatal care, and MMD. Previous studies in Eastern and Southern Africa have found associations between all these factors and IIT in their context [5, 7–9, 16–18]. However, none assessed MMD. While we evaluated IIT, which we defined as missing the first ART follow-up visit by greater than 28 days after enrollment in PVT services, prior studies that we refer to all used different definitions of IIT, lost to follow-up, retention in care, or continuity of treatment.

This analysis demonstrated that, overall, among the study population, 37.2% of participants with PBFW had IIT. This falls within the upper range of estimates from a 2018 review of eight African countries, which estimated IIT after the first PVT visit to be between 3% and 44% [5].

PBFW in South Sudan receive HIV treatment in ART clinics separately from antenatal and postnatal care. Our study found that only 21.8% of pregnant women visited ANC clinics three or more times during their current pregnancy, well below the WHO guidelines adopted by South Sudan, which recommended eight ANC contacts through pregnancy [19]. Pregnant women living with HIV who attended only one ANC visit were 20% less likely to interrupt treatment compared to pregnant women who attended three or more ANC visits during their current pregnancy. Although this finding was unexpected, a possible explanation is that the low number of ANC visits among pregnant women on ART and the lack of alignment between ANC and ART clinic appointments observed during follow-up visits, PBFW in this study, are possibly choosing to prioritize ART visits over ANC visits, given the constraints of attending both. In clinics, half of the women in this analysis only attended one ANC visit, promoting health education and the importance of both ART and ANC visits. Reaching women who do not access the health system through community engagement and programming may improve their access to healthcare services. Aligning HIV clinic dates with ANC visit dates and fully integrating ANC and PVT services can help increase attendance at both clinics.

Despite efforts to reduce maternal and child mortality through the promotion of facility-based delivery by skilled attendants [19], many women in South Sudan deliver their babies at home. The United Nations cooperation framework estimates indicate that 87% of deliveries in South Sudan occur at home, and a 2020 household survey in South Sudan estimated that 58% of women gave birth at home during their last pregnancy [12]. While the home delivery rate for women living with HIV in our study was high at 23.6%, it is lower than previous estimates for the general population of pregnant women in South Sudan. In our study, home delivery was associated with IIT among pregnant women. These findings support focusing interventions on women who say they may not give birth in a health facility or who have in the past given birth at home. Interventions that may be helpful include education around the benefits of facility delivery, addressing access barriers, like transportation, to general antenatal care, and ensuring continuity in HIV care for pregnant women in South Sudan.

This is one of the few studies to evaluate MMD in pregnant women, as most studies exclude pregnant women with MMD from their analysis. A survey conducted in the general population of South Sudan found that providing 3–6 months of MMD was feasible and supported client retention (Kieran Hartsough HA, Shambel, Aragaw FB, Altaye Kidane, Onyekachi, Ukaejiofo HS, Semira M, Najohapa J: SMS: Rapid Expansion of Multi Month Dispensing to Support Retention of People Living with HIV on Antiretroviral Therapy during the COVID 19 Pandemic in South Sudan, unpublished). Another study in the general population in Zambia and Malawi found that retention in care at 12 months (defined as less than 60 consecutive days without ART) was non-inferior for people who received ≥ 6 MMD, compared to 3 MMD and no MMD (Kieran Hartsough HA, Shambel, Aragaw FB, Altaye Kidane, Onyekachi, Ukaejiofo HS, Semira M, Najohapa J: SMS: Rapid Expansion of Multi Month Dispensing to Support Retention of People Living with HIV on Antiretroviral Therapy during the COVID 19 Pandemic in South Sudan, unpublished) [20]. South Sudan’s national guidelines do support MMD for pregnant women, given the political instability, conflict, and severe droughts and floods. In our study, most pregnant women (59.8%) were given ≥ 6 months of MMD during their first follow-up visit. Those who received less than 3 months of MMD were 1.6 times more likely to experience interruption in treatment compared to those who received 6 or more months of MMD. This supports the continued use of MMD for pregnant women for at least 6 months, particularly in conflict and environmental disaster zones. In these areas, women may face challenges attending clinic appointments, making it crucial for them to have access to their medication for an extended period. In South Sudan, the ANC appointments are separate from the ART clinic appointments. This means that women who have received ≥ 6 months of MMD are not expected to come in for their ART refills before 6 months have passed. However, they are expected to have more frequent ANC appointments. Because ART appointments are not always scheduled on the same day as ANC appointments, receiving a ≥ 6-month MMD reduces additional transportation costs and clinic waiting time. When the ANC and ART appointments are scheduled on the same day, women would still need to wait in two separate queues. Receiving a ≥ 6-month MMD can save clients time and money.

While other studies have found a significant association between client residence and IIT [21–23], ours did not. In our study, approximately one-third of women from each of the three areas (urban, peri-urban, and rural) reported IIT. This may be attributed to the classification of geographical areas. For instance, in South Sudan, there are no major differences between rural and peri-urban areas in terms of health infrastructure and health service delivery. However, transportation issues may be present in all areas and have been shown to contribute to IIT [24]. While previous studies in Africa have reported that age (younger and older age groups) is associated with treatment interruption [9, 16, 24–26], we did not find any association with these factors. Our study also did not align with previous findings that PBFW newly diagnosed with HIV are more likely to interrupt treatment [5, 27].

We acknowledge several study limitations. First, programmatic data sources from health facilities may contain missing and inaccurate data. To mitigate this, we used multiple data sources to identify the relevant variables from other data sources to determine if they were missing from one data source. Second, we calculated MMD by using a proxy based on appointment dates: the number of days between the date of the initial visit and the date of the follow-up visit. This was done with the assumption that the prescriber gave a follow-up appointment date based on the amount of medication prescribed. Third, some transfers to other facilities may not have been correctly recorded and may have been misclassified as IIT. Fourth, the data analysis did not include a longitudinal component to assess multiple instances of IIT. Fifth, The South Sudan national treatment guideline recommends 6-month MMD for all adult clients regardless of whether the patients are stable. Persons who are given a shorter duration of MMD may be those at higher risk of IIT, either judged by a clinician, or they may be at risk for other reasons, such as Advanced HIV Disease (AHD) and the need for frequent monitoring of their viral load. However, PBFW given at least 6 moths of MMD faired no worse, thus MMD is not a risk factor for IIT in the context of South. Sixth, all the facilities included in this study were supported by PEPFAR and had same standards of care and support levels. However, differences did exist particularly between urban and rural settings. Due to the nature of the data, we did not include this information in the analysis. Finally, this study is not generalizable beyond the context of South Sudan.

## Conclusion

Despite significant investment in rapid ART initiation for PBFW and PVT services, interruption in treatment among PBFW on ART remains a challenge in South Sudan. Over one-third of PBFW on ART had IIT in our study. Delivery of an infant at home and receiving fewer months of ARV medications were significantly associated with IIT, while women who attended only one ANC visit were less likely to interrupt treatment. Integration of ANC and ART services may improve continuity of HIV care and treatment and reduce the burden on women attending multiple appointments. The unique practice in South Sudan of prescribing six months of ART for PBFW seemed protective of IIT. While there are data limitations, this indicates that longer MMD for PBFW may be an effective option for other countries facing similar barriers. Further work should focus on exploring interventions that enhance community engagement and increase demand for antenatal care, such as client tracing through the mentor-mothers, and community outreach volunteers to support adherence to ART appointments. In addition, future studies should evaluate viral load suppression and outcomes for HIV-exposed infants in relation to MMD. Addressing both health system and community-level barriers with context-adapted strategies, such as MMD, integrated ANC and ART services, and strengthened community-based support, could be essential for improving retention and reducing treatment interruption among pregnant and breastfeeding women living with HIV in South Sudan.

## Supplementary Information

Additional file 1: Data Used to replicate findings in the manuscript (XLSX 680 kb).

Additional file 2: Data Abstraction form (docx 21 kb).

## Data Availability

I have the raw data used to generate findings for this research article.

## Abbreviations

ANC: antenatal care
ART: antiretroviral therapy
CDC: U.S. Center for Disease Control and Prevention
DGHT: Division on Global HIV and Tuberculosis
IIT: Interruption in Treatment
MMD: Multi-month dispensing
PBFW: Pregnant and breastfeeding women
PEPFAR: U.S. President’s Emergency Plan for AIDS Relief
PVT: Prevention of Vertical Transmission
WHO: World Health Organization
