# Supplementary material for: Factors associated with interruption in treatment among pregnant and breastfeeding women living with HIV on ART in South Sudan
Source: BMC Public Health. 2026 Apr 15;26:1685. doi: 10.1186/s12889-026-27413-1 (PMC13200411; doi:10.1186/s12889-026-27413-1)
Supplement: Supplementary file 2 — Additional file 2: Data Abstraction form (docx 21 kb). [file 12889_2026_27413_MOESM2_ESM.docx]

# Appendix II

# Data Abstraction Form

**Health Facility Data Abstraction Tool: Pregnant and Breastfeeding Women Enrolled in PMTCT Care**

Maternal Data Abstraction ID: ______________________Date of Abstraction: ___________________________

Abstracted by: ______________________________Data check conducted by: ____________________________

**Name of health facility: ______________________**

**SOCIO-DEMOGRAPHIC CHARACTERISTICS:**

| **DATA SOURCE: ANC/PMTCT REGISTER** | |
| --- | --- |
| **1. Date of ANC Registration (Column 2)** |  |
| **2. Age of woman at ANC Registration** *(years)* **(Column 12)** |  |
| **DATA SOURCE: ANC Register** | |
| **3. Name of Village (Column 7)** |  |
| 1. **Marital status (Column 10)** | 🗖Single (never married)  🗖Married Monogamous  🗖Married Polygamous  🗖 Divorced/separated  🗖Widowed |
| 1. **Main Occupation (Column 9)** | 🗖1. Unemployed  🗖2. Student  🗖3. Housewife  🗖4. Military Personnel  🗖**5.** Salaried Employee  🗖 **6.** Other Uniformed forces (Police, Wildlife, Prisons, Fire Brigade)  🗖**7.** Farming  🗖**8.** Business (Man/Women)/trader  🗖**9.** All other professions |

**PATIENT’S OBSTETRIC HISTORY AND CURRENT PREGNANCY**

| **DATA SOURCE: ANC REGISTER** | | |
| --- | --- | --- |
| 1. **Para (Column 17)** | 🗖 1 🗖 2 🗖3 or more | |
| 1. **Gravida (Column 17)** | 🗖 1 🗖 2 🗖 3 or more | |
| **DATA SOURCE: ANC/PMTCT register** | | |
| 1. **EDD (column 15) [**DD/MM/YYYY] |  | |
| 1. **Gestational age at ANC registration** *(weeks)* **(column 16)** |  | |
| 1. **Entry to PMTCT (column 6)** | 🗖Newly tested at ANC  🗖Known Pos on ART  🗖Known Pos not on ART | |
| 1. **Number of ANC visits (count visits prior to Date of Delivery)** | 🗖 1 visit 🗖 2 visits 🗖3 visits 🗖 4 or more visits | |
| 1. **Place of delivery (column 32)** | 🗖 At Health Facility 🗖 At home | |
| 1. **Delivery outcome (Column 33)** | 🗖 Live Birth-LB  🗖 Still Birth-MSB or FSB  🗖 Neonatal Death-ND | |
| 1. **Treatment status and timing (Columns 7, 10, 31)** | | 🗖Already on ART  🗖 New on ART -- in pregnancy (ART initiated during pregnancy but prior to date of delivery)  🗖 New on ART -- postpartum (ART initiated after date of delivery) |
| 1. **Date of ART Initiation** *(dd/mm/yy)* **(Column 10)** | |  |

**TREATMENT CONTINUITY [DATA SOURCE: ANC/PMTCT REGISTER; ART PATIENT CARD (YELLOW CARD)]**

1. **Client Appointment information.** *Record visit dates for PMTCT care in Column B from the ANC/PMTCT Register up to 8 visits maximum. Then, refer to ART card to fill out additional columns on follow up date, drug dispensing, and VL.*

| Visit number  [Starting Oct 1, 2019] | Visit date | Pregnant/ Breastfeeding | Follow up date | Number of days of ART dispensed | VL (Y/N) | VL result |
| --- | --- | --- | --- | --- | --- | --- |
| [A] | [B] | [C] | [D] | [E] |  |  |
| Visit 1 |  | Preg Bf |  |  |  |  |
| Visit 2 |  | Preg Bf |  |  |  |  |
| Visit 3 |  | Preg Bf |  |  |  |  |
| Visit 4 |  | Preg Bf |  |  |  |  |
| Visit 5 |  | Preg Bf |  |  |  |  |
| Visit 6 |  | Preg Bf |  |  |  |  |
| Visit 7 |  | Preg Bf |  |  |  |  |
| Visit 8 |  | Preg Bf |  |  |  |  |
